# Supplementary material for: Vitamin D Modified DSS-Induced Colitis in Mice via STING Signaling Pathway
Source: Biology (Basel). 2025 Jun 18;14(6):715. doi: 10.3390/biology14060715 (PMC12190092; doi:10.3390/biology14060715)
Supplement: Supplementary file 1 [file biology-14-00715-s001.zip › Supplementary Materials S1: 16s-report of gut microbiota/Groups/diff_analysis/TukeyHSD/Tukey_Bugbase.pdf]

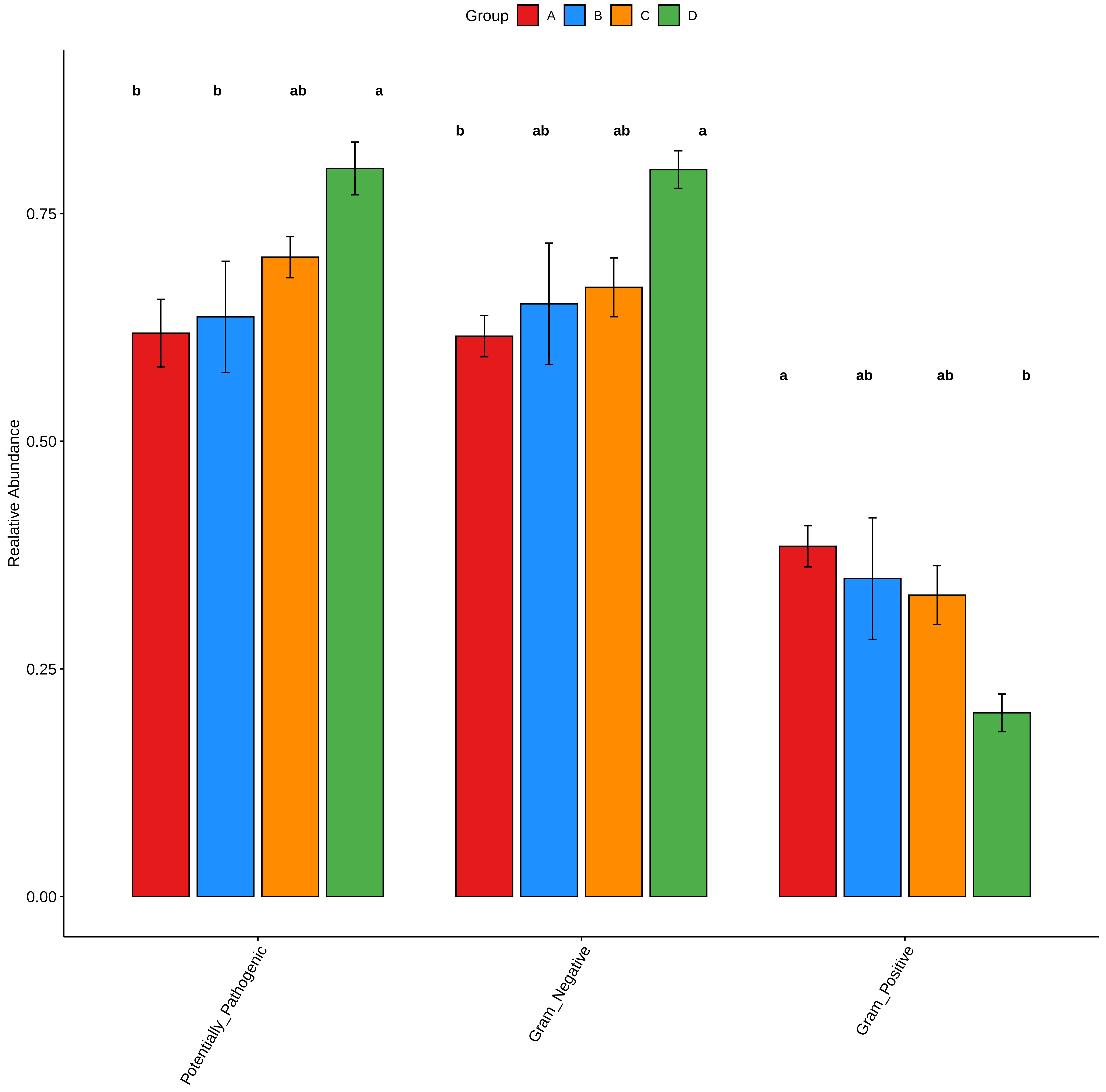
Group
A
B
C
D
b
b
ab
a
b
ab
ab
a
a
ab
ab
b
Realative Abundance
0.75
0.50
0.25
0.00
Potentially_Pathogenic
Gram_Negative
Gram_Positive
